# Draft Genome Sequence of *Listeria monocytogenes* Strain CIIMS-PH-1, a Serovar 4b Isolate from Infant Septicemia

**DOI:** 10.1128/genomeA.01320-17

**Published:** 2018-02-15

**Authors:** Pooja M. Kishnani, Ankita A. Tiwari, Vikas Gautam, Megha Sharma, Sukhadeo B. Barbuddhe, Swapnil P. Doijad, Trinad Chakraborty, Amit R. Nayak, Nidhi M. Bhartiya, Hatim F. Daginawala, Lokendra R. Singh, Rajpal S. Kashyap

**Affiliations:** a Biochemistry Research Laboratory, Central India Institute of Medical Sciences (CIIMS), Nagpur, India; b Postgraduate Institute of Medical Education and Research, Chandigarh, India; c ICAR-National Research Centre on Meat, Hyderabad, India; d Institute of Medical Microbiology, Biomedizinisches Forschungszentrum Seltersberg, Giessen, Germany

## Abstract

We report here the draft genome sequence of *Listeria monocytogenes* CIIMS-PH-1, an isolate obtained from a 16-day-old infant with septicemia. The draft genome of CIIMS-PH-1 consisted of 2,939,183 bp and is a member of sequence type 308, clonal complex 1, and lineage I.

## GENOME ANNOUNCEMENT

*Listeria monocytogenes* is a facultative, anaerobic, Gram-positive pathogenic bacterium that causes gastrointestinal infection, meningitis, septicemia, abortion, and in some cases death, in humans as well as in livestock (1). *L. monocytogenes* infects a broad population range, especially immunocompromised individuals such as newborns, the elderly, cancer patients, and pregnant women (2). Among the 13 serotypes of *L. monocytogenes*, strains belonging to serotypes 4b, 1/2a, and 1/2b have been primarily associated with infections (2).

Here, we report the draft genome sequence of the clinical isolate *L. monocytogenes* strain CIIMS-PH-1, which was isolated from blood samples of a 16-day-old infant diagnosed with septicemia, from Chandigarh, a city in North India. To understand the properties of this isolate comprehensively, we performed in-depth whole-genome sequencing. Using a Qiagen genomic DNA extraction kit, genomic DNA was isolated from culture freshly grown on brain heart infusion agar. Libraries were constructed by using a paired-end library (2 × 100-bp) reagent kit. The sequencing of the isolate was performed on an Illumina HiSeq 2500 platform. Reads were filtered with Trimmomatic (3), and *de novo* assembly was carried out using SPAdes version 3.10.1 (4). Annotations were carried out using Prokka (5).

The draft genome of isolate CIIMS-PH-1 consisted of 2,939,173 bp with a 615-fold overall coverage. The genome had a GC content of 37.94% as well as 2,975 genes, including 2,843 coding sequences, 67 tRNAs, 6 rRNAs, and 43 pseudogenes. CIIMS-PH-1 showed a 95% average nucleotide identity with the reference strain *L. monocytogenes* EGDe, reconfirming its taxonomic allocation. Further genome analysis shows the presence of the *Listeria* pathogenicity island I (LIPI-I) and other earlier reported virulence genes. *In silico* multilocus sequence typing identified isolate CIIMS-PH-1 as a member of sequence type 308, clonal complex 1, and lineage I. A BLAST search of the contigs against the NCBI plasmid database did not reveal any plasmid contigs. Analysis of isolate CIIMS-PH-1 with a SEED-based subsystem classification through the Rapid Annotations using Subsystems Technology (RAST) server (RAST genome number 6666666.284991) revealed *L. monocytogenes* isolate ScottA to be its closest phylogenic neighbor (score of 545). In addition, the TEM-116 gene-encoding extended-spectrum β-lactamases (ESBLs), which confer resistance to the commonly used β-lactam antimicrobials and ESBL-producing bacteria that render treatment difficult in human and veterinary medicine, were found (6). These are common characteristics found in bacteria belonging to the family *Enterobacteriaceae*.

Unlike in developed countries, the incidences of *L. monocytogenes*, particularly from clinical cases, are highly underreported among developing countries such as India. Moreover, only a few of their genomes have been sequenced and are available publicly. Nevertheless, the availability of genomes of such isolates is crucial to understanding the distribution and trafficking of strains worldwide. This genome will be further studied to determine the characteristics of the strain and for comparative analysis.

### Accession number(s).

The annotated whole-genome sequence of *L. monocytogenes* strain CIIMS-PH-1 has been deposited in GenBank under accession number CP023321.

## References

[1] Buchanan RL, Gorris LGM, Hayman MM, Jackson TC, Whiting RC 2017 A review of *Listeria monocytogenes*: an update on outbreaks, virulence, dose-response, ecology, and risk assessments. Food Control 75:1–13. doi:10.1016/j.foodcont.2016.12.016.

[2] Swaminathan B, Gerner-Smidt P 2007 The epidemiology of human listeriosis. Microbes Infect 9:1236–1243. doi:10.1016/j.micinf.2007.05.011. 17720602

[3] Bolger AM, Lohse M, Usadel B 2014 Trimmomatic: a flexible trimmer for Illumina sequence data. Bioinformatics 30:2114–2120. doi:10.1093/bioinformatics/btu170. 24695404 PMC4103590

[4] Bankevich A, Nurk S, Antipov D, Gurevich AA, Dvorkin M, Kulikov AS, Lesin VM, Nikolenko SI, Pham S, Prjibelski AD, Pyshkin AV, Sirotkin AV, Vyahhi N, Tesler G, Alekseyev MA, Pevzner PA 2012 SPAdes: a new genome assembly algorithm and its applications to single-cell sequencing. J Comput Biol 19:455–477. doi:10.1089/cmb.2012.0021. 22506599 PMC3342519

[5] Seemann T 2014 Prokka: rapid prokaryotic genome annotation. Bioinformatics 30:2068–2069. doi:10.1093/bioinformatics/btu153. 24642063

[6] Jiang X, Zhang Z, Li M, Zhou D, Ruan F, Lu Y 2006 Detection of extended spectrum β lactamase in clinical isolates of *Pseudomonas aeroginosa*. Antimicob Agents Chemother 9:2990–2995. doi:10.1128/AAC.01511-05. PMC1563526 16940093

